# Study of Sexual Satisfaction in Different Typologies of Adherence to the Sexual Double Standard

**DOI:** 10.3389/fpsyg.2020.609571

**Published:** 2021-01-08

**Authors:** Ana Álvarez-Muelas, Carmen Gómez-Berrocal, Juan Carlos Sierra

**Affiliations:** Mind, Brain and Behavior Research Center, University of Granada, Granada, Spain

**Keywords:** sexual satisfaction, sexual double standard, typologies, predictors, gender

## Abstract

The sexual double standard (SDS) refers to the acceptance of different criteria to assess the same sexual behavior in men and women. To date, the few studies that have addressed the relationship between SDS and sexual satisfaction have obtained inconclusive results. In addition, no study has analyzed sexual satisfaction in people who maintain different forms of adherence to the SDS. This study establishes three SDS typologies of adherence (man-favorable, woman-favorable, egalitarian) in two areas of sexual behavior (sexual freedom and sexual shyness) to examine the predictive capacity of personal variables (age, social dominance orientation, propensity for sexual excitation/inhibition), interpersonal variables (relationship satisfaction) and social variables (gender norms about sexual behaviors) in sexual satisfaction. A sample of 1194 heterosexual adults (51.1% men, 48.8% women) aged between 18 and 87 years (*M* = 40.63; *SD* = 15.67), who had been in a relationship for more than 6 months, was evaluated. In men, the highest sexual satisfaction levels were obtained in the egalitarian typology in the sexual freedom area. In women, no significant differences were found between the typologies of adherence to the SDS. Regression models showed that relationship satisfaction was the main predictor of sexual satisfaction in all the typologies in both men and women. In addition, the predictive relationship of personal variables with sexual satisfaction varied according to gender and the SDS adherence type. The results show the importance of studying sexual satisfaction by taking into account not only the differences between men and women. Furthermore, it is essential to consider other differences between people; for example, the difference that derives from the way of psychologically internalizing attitude toward the SDS.

## Introduction

One of the most relevant manifestations of sexual health is sexual satisfaction (Henderson et al., 2009; World Health Organization., 2010), which suggests a subjective dimension of sexuality and is defined as “an affective response arising from one’s subjective evaluation of the positive and negative dimensions associated with one’s sexual relationship” (Lawrance and Byers, 1995, p. 268). Sexual satisfaction does not depend only on one’s own sexual relationships, but also on other personal, interpersonal, and socio-cultural factors (Sánchez-Fuentes et al., 2014; Calvillo et al., 2018); that is, studying it requires multicomponent models. For that purpose, Henderson et al. (2009); Sánchez-Fuentes et al. (2016), and Calvillo et al. (2020a) based their works on the Ecological Theory of Human Development (Bronfenbrenner, 1994), which conceives personal development as the result of the interaction of individuals with the environmental contexts in which they live and socialize. The most relevant factors in these models for explaining sexual satisfaction are those of the personal and interpersonal kind (Sánchez-Fuentes et al., 2014; Calvillo et al., 2018).

A personal factor that has been associated with sexual satisfaction, albeit with inconsistent results to date, is the sexual double standard (SDS); that is, the attitude which involves a distinct evaluation of given sexual behaviors depending on if they are performed by a man or a woman, where men have more freedom and/or permissibility than women (Álvarez-Muelas et al., 2020b). Some studies report a negative association between the SDS that favors men and sexual satisfaction (Haavio-Mannila and Kontula, 2003; Horne and Zimmer-Gembeck, 2006; Santos-Iglesias et al., 2009), while others have found no relation between both variables (Marques et al., 2013). We believe that there might be several reasons for this inconsistency in using measures that do not accurately capture sexual satisfaction, and for not considering some individual differences that could play a relevant role in the sexual satisfaction relationship. As regards the sexual satisfaction measure, isolated questions are normally employed (e.g., Haavio-Mannila and Kontula, 2003), or scales that mix sexual satisfaction items with items about sexual satisfaction-related variables, such as sexual attitude, desire or sexual excitation (e.g., Santos-Iglesias et al., 2009; Marques et al., 2013). We believe that this limitation can be overcome by using the Global Measure of Sexual Satisfaction, which has been validated in a Spanish population (Sánchez-Fuentes and Santos-Iglesias, 2016), and is one of the measures included in the Interpersonal Exchange Model of Sexual Satisfaction Questionnaire (Sánchez-Fuentes et al., 2015; Calvillo et al., 2020b; Lawrance et al., 2020). This evaluation instrument is based on one of the few theoretical models of sexual satisfaction (Lawrance and Byers, 1992, 1995), and is the only one that has been validated in a Spanish population (Sánchez-Fuentes and Santos-Iglesias, 2016).

On the other hand, we are interested in knowing if individual differences in SDS are related to sexual satisfaction. In accord with Endendijk et al. (2020), to analyze the role of individual differences in attitude toward SDS, we consider the degree to which people have internalized SDS in their own social cognitions. From this approach, we study sexual satisfaction taking into account the different forms of adherence to SDS. Previous studies show that various forms of adherence to SDS prevail, from the attitude that favors men to that which favors women (Álvarez-Muelas et al., 2020b; Endendijk et al., 2020). In addition, the prevalence of forms of adherence to SDS differs in the field of behavior related to sexual freedom (SF; that is, “the recognition and approval of the benefit for men and women, of freely having sex and respecting sexual rights”), and in the area of sexual shyness (SS; that is,“the recognition and approval of men and women’s will to manifest decorum, chastity, and continence in sexual relations”) (Álvarez-Muelas et al., 2020b, p. 2).

Moreover, in order to understand sexual satisfaction, the proposal of personal, interpersonal, and social factors could be considered in accordance with the Ecological Theory (Bronfenbrenner, 1994). In the present study, we kept some variables with previous evidence with respect to sexual satisfaction (age, propensity for sexual excitation/inhibition, relationship satisfaction, and gender norms about sexual behaviors), and we included a variable would play a relevant role according to the different form of adherence to SDS (social dominance orientation).

Age stands out among the personal variables, which has been negatively associated with sexual satisfaction (Tren and Schaller, 2010; De Ryck et al., 2012; Sánchez-Fuentes and Sierra, 2015; Træen et al., 2017; Wyverkens et al., 2018).

Another personal factor that has not yet been considered to date is social dominance orientation. Social dominance orientation is an individual characteristic that predisposes someone to support and defend a social structure, where intergroup relations (e.g., between men and women) are hierarchical and non-egalitarian (Sidanius and Pratto, 1999). There is evidence to suggest that those people who manifest social dominance orientation support discriminating ideologies toward women’s rights (Pratto et al., 1994), the traditional gender roles (Christopher and Wojda, 2008) and, concretely, the SDS that favors men in both the sexual freedom and sexual shyness areas (Sierra et al., 2018). Moreover, social dominance orientation tends to support discriminating toward men and women’s sexual behaviors (Kelly et al., 2015). From such this perspective, social dominance orientation could be relevant for predicting sexual satisfaction considering the SDS adherence type, especially in those people who belong to an SDS adherence type in agreement with the hegemony of one gender category over another (i.e., man-favorable typology and woman-favorable typology).

We also considered the propensity for sexual excitation/inhibition, proposed with the Dual Control Model (Janssen and Bancroft, 2007). What this model assumes is that people possess an excitation system, as well as another system that inhibits the sexual response and associated behaviors. The inhibitory system contains two subsystems: inhibition due to threat of performance failure and inhibition due to threat of performance consequences (Janssen et al., 2002a,b; Carpenter et al., 2008). According to former findings, sexual satisfaction was associated negatively with to sexual excitation (Lykins et al., 2012), inhibition due to threat of performance failure (Lykins et al., 2012; Moyano and Sierra, 2014; Arcos-Romero and Sierra, 2020), and inhibition due to threat of performance consequences (Moyano and Sierra, 2014).

Of the interpersonal factors, relationship satisfaction has been demonstrated to be the most important one as a determining factor of sexual satisfaction (Byers, 2005; Sánchez-Fuentes et al., 2015; Sánchez-Fuentes and Santos-Iglesias, 2016; Calvillo et al., 2020a).

Finally, it based on the assumption that social norms predict conduct (Cialdini et al., 1990), it was demonstrated that the way sexual norms are perceived can influence subjective feelings of sexual satisfaction (Stephenson and Sullivan, 2009). Despite the fact that gender norms in sexual relations can confer women less power because gender roles dictate feminine submission and masculine dominance (Impett and Peplau, 2003; Kiefer et al., 2006), evidence suggests that being involved in adhering to gender norms can negatively affect sexual satisfaction in both men and women (Sánchez et al., 2005). Faced with this evidence, we explored not only those individuals who maintained different SDS adhesion, but also the perception of gender norms about sexual behaviors.

Therefore, based on the Ecological Theory of Human Development framework (Bronfenbrenner, 1994), this study considers the different forms of individual SDS adherence to examine the sexual satisfaction relationship with personal (age, social dominance orientation, propensity for sexual excitation/inhibition), interpersonal (relationship satisfaction) and social (gender social norms about sexual behavior) factors.

When considering gender (i.e., men and women) and SDS adherence types in the SF and SS areas:

RQ1: Would there be any differences in sexual satisfaction and related variables of personal (age, social dominance orientation, and propensity for sexual excitation/inhibition), interpersonal (relationship satisfaction) and social (gender norms about sexual behaviors)?
RQ2: What predictive capacity would personal (age, social dominance orientation, and propensity for sexual excitation/inhibition), interpersonal (relationship satisfaction), and social (gender norms on sexual behaviors) variables have on sexual satisfaction?

## Materials and Methods

### Participants

The sample was made up of 1194 adults (610 men, 584 women) aged between 18 and 87 years (*M* = 40.63; *SD* = 15.67) and recruited by non-random sampling. The inclusion criteria included: (1) Spanish nationality; (2) heterosexual orientation; (3) being 18 years old or older; (4) being involved in a heterosexual relationship for at least 6 months. Significant gender differences were found in the sample. Men reported having more sexual partners (*t* = 2.99; *p* < 0.005) and younger partners (*t* = −3.02; *p* < 0.005). Table 1 presents the sample’s socio-demographic characteristics.

**TABLE 1:** Sociodemographic characteristics of the participants.

Variables	Total (*N* = 1194)	Men (*n* = 610)	Women (*n* = 584)		

	*M* (*SD*)	*M* (*SD*)	*M* (*SD*)	*t/*χ ^2^	Cohen’s *d*
Age	40.63 (15.67)	41 (15.36)	40.25 (15.99)	0.83	
Age of the first sexual experience	18.15 (3.66)	17.91 (3.67)	18.41 (3.64)	–2.33	–0.14
Number of sexual partners	4.80 (11.12)	5.75 (14.72)	3.82 (5.15)	2.99*	0.18
Partner age	40.56 (15.56)	39.21(14.75)	41.95 (16.26)	−3.02*	–0.18
Length of the relationship (years)	16.60 (14.55)	16.14 (14.08)	17.08 (15.03)	–1.07	
Education level				1.42	
No studies	4.9%	4.4%	5.3%		
Primary school	15.7%	14.8%	16.6%		
High school	28.6%	29.2%	27.9%		
University	50.9%	51.6%	50.2%		

*M*: mean; *SD*: standard deviation; *t*: Student’s; χ^2^: chi-square. *p < 0.005.

### Measurements

– Socio-demographic and Sexual History Questionnaire. We designed a questionnaire to assess gender, age, nationality, sexual orientation, sexual activity in the relationship, partner’s age, length of the relationship, age of first sexual experience, number of sexual partners and level of education.
– Spanish version of the Sexual Double Standard Scale (SDSS; Muehlenhard and Quackenbush, 2011; Sierra et al., 2018). The scale is a self-referred measure of the SDS. It consists of 16 items answered on a 4-point Likert-type scale from 0 (*strongly disagree*) to 3 (*strongly agree*), and distributed into two factors: Acceptance of sexual freedom and Acceptance of sexual shyness. Each factor is formed by four pairs of parallel items: one refers to sexual behavior attributed to men, and the other to sexual behavior attributed to women. The responses to Acceptance of sexual freedom allow the Index of Double Standard for Sexual Freedom (IDS-SF) to be obtained, and the responses to the Acceptance of sexual shyness items allow the Index of Double Standard for Sexual Shyness (IDS-SS) to be acquired. Both indices represent a bipolar measurement (between −12 and +12). The man-favorable typology includes those people with positive scores in the index (between +1 and +12). The woman-favorable typology is obtained from the scores that take a negative value (between −1 and −12). Finally, the egalitarian typology includes those people whose score equals zero in either index and obtain a zero result in the subtractions between the pairs of parallel items of the index. The scale suitably evidenced internal consistency (Cronbach’s ordinal alpha 0.84 for the Acceptance of sexual freedom factor and 0.87 for the Acceptance of sexual shyness factor) (Sierra et al., 2018). It was invariant for gender and age (by eliminating the pair of items 11 and 14 which showed DIF by age) (Álvarez-Muelas et al., 2019). So these pairs of items were removed from the present study. The ordinal alphas were respectively 0.89 and 0.91 for the Acceptance of sexual freedom factor, and 0.87 and 0.89 for the Acceptance of sexual shyness factor, in men and women.
– The Spanish version of Social Dominance Orientation Scale (SDOS; Pratto et al., 1994; Silván-Ferrero and Bustillos, 2007). It consists of 16 items that are answered on a 7-point Likert scale from 1 (*completely disagree*) to 7 (*completely agree*), and two factors: General opposition to equality and Support for group-based dominance (Jost and Thompson, 2000; Silván-Ferrero and Bustillos, 2007). Whereas General opposition to equality is conceived as justifying the hierarchical social system, Support for group-based dominance is defined as a way to justify the own group’s dominance (in-group) (Jost et al., 2004). Cronbach’s alpha coefficients were 0.84 for men and 0.77 for women. In this study, the ordinal alpha coefficients were 0.90 in men and 0.91 in women for General opposition to equality, and 0.72 in men and 0.82 in women for Support for group-based dominance.
– The Spanish version of the Sexual Inhibition/Sexual Excitation Scales-Short Form (SIS/SES-SF; Carpenter et al., 2011; Moyano and Sierra, 2014). This scale evaluates the individual propensity for sexually excited or inhibited. Its 14 items are answered on a 4-point Likert-type scale from 1 (*strongly agree*) to 4 (*strongly disagree*). The items are distributed into three subscales: Sexual excitation, Inhibition due to threat of performance failure, Inhibition due to threat of performance consequences. Cronbach’s alpha coefficients range between 0.60 and 0.72. In this sample, the ordinal alpha values range fell between 0.73 and 0.79 for men and between 0.68 and 0.82 for women.
- The Spanish version of Global Measure of Relationship Satisfaction (GMREL; Lawrance et al., 2011; Sánchez-Fuentes et al., 2015). It evaluates satisfaction with partner relationship using five seven-point bipolar subscales: *Very bad/Very good; Very unpleasant/Very pleasant; Very negative/Very positive; Very unsatisfying/Very satisfying; Very worthless/Very valuable*. Its Cronbach’s alpha coefficients are 0.94 for men and women (Sánchez-Fuentes et al., 2015). In the present study, the ordinal alpha values were 0.94 for men and 0.96 for women.
– The Spanish hetero-referred version of Sexual Double Standard Scale (SDSS-H; Muehlenhard and Quackenbush, 2011; Gómez-Berrocal et al., 2019). It evaluates the subjective perception of gender norms about sexual behaviors. The scale is composed of 18 items answered on a 4-point Likert-type scale from 0 (*strongly disagree*) to 3 (*strongly agree*), and three factors: Acceptance of sexual shyness in men, Acceptance of sexual freedom in women, Acceptance of traditional gender role distribution. For each factor, the internal consistency obtained ordinal alpha values that equal 0.73, 0.70, and 0.90, respectively. In this sample and for each factor, the values were 0.72, 0.68, and 0.86 for men, and 0.75, 0.67, and 0.91 for women.
– The Spanish Global Measure of Sexual Satisfaction (GMSEX; Lawrance et al., 2011; Sánchez-Fuentes et al., 2015). It evaluates overall sexual satisfaction in a relationship using five seven-point bipolar subscales: *Very bad/Very good; Very unpleasant/Very pleasant; Very negative/Very positive; Very unsatisfying/Very satisfying; Very worthless/Very valuable*. Its Cronbach’s alpha coefficients are 0.92 for men and 0.93 for women (Sánchez-Fuentes et al., 2015). In this sample, its ordinal alpha values were 0.94 for men and 0.95 for women.

### Procedure

The study was previously approved by the Human Research Ethics Committee of the University of Granada. The target population was defined in the inclusion criteria of the study. Participants were recruited from the general Spanish population by incidental sampling to obtain a balanced proportion of men and women, and also across age groups (18–34; 35–49; 50 years old or older), between March 2018 and February 2019. The evaluation in the paper-and-pencil format (86.6% of the sample) and the online format (13.4% of the sample) was used. Both procedures showed no differences in the responses in terms of information on general behaviors (Carreno et al., 2020) or sexual behaviors (Sierra et al., 2018). The evaluation format presented low or non-existent correlations with the other analyzed variables. The participants who completed questionnaires in paper and pencil format were approached using snowball sampling techniques in educational, community, and leisure centers. Firstly, we requested the approval of the center, which was informed on the objective of the research. The questionnaires were managed by a trained evaluator, and the participants answered in small groups or individually, which were returned in sealed envelopes. The online questionnaires were created on the LimeSurvey platform. The URL to access was distributed through social networks (Facebook^®^, Twitter^®^, WhatsApp^®^ groups, and e-mail). The IP address was controlled and automatic responses were avoided by answering a security question consisting of a random arithmetic question. The participants accepted an informed consent form which specified the overall objective of the study. Anonymity and confidentiality were guaranteed, and their participation was voluntary without compensation.

### Data Analysis

First, gender differences by a Student’s *t*-test for two independent groups (i.e., men and women) were calculated for the scores of the SDS indices for Sexual Freedom (ID-SF) and Sexual Shyness (IDS-SS). The results revealed gender differences in both the IDS-SF (*t* = 5.22; *p* < 0.001) and the IDS-SS (*t* = 6.03; *p* < 0.001). Due to the found differences, we decided to divide the sample into men and women separately. According to the scores of the indices, the sample of men and women was distributed into the SDS adherence types (egalitarian, man-favorable, woman-favorable) for the SF and SS areas. Second, by using an ANOVA for men and women, the differences for all the variables were calculated by typologies of adherence to SF and SS. Finally, we examined the degree to which sexual satisfaction could be explained by the different variables (personal, interpersonal, social) with multiple linear regression using the stepwise method for each SDS typology of both sexual behavior areas. Independent variables were included in each step according to the significance of their correlation with sexual satisfaction. The degree of multicollinearity was assessed with the tolerance value and the variance inflation factor (VIF). When the tolerance value was >0.10 and the VIF was <10 for the predictor variables, there were no serious problems with multicollinearity (López, 1998; Dormann et al., 2013; Lavery et al., 2017). A *p*-value of 0.005 represented significant differences. This range indicates evidence according to conventional Bayes factor classifications and can reduce the probability of type I errors (Benjamin et al., 2018).

## Results

### Differences in Sexual Satisfaction and Associated Variables by SDS Adherence Type and Sexual Behavior Area

In men, significant differences were found in sexual satisfaction between the different SDS adherence types in SF (*F* = 8.41; *p* < 0.001), with higher scores for the egalitarian typology than for the man-favorable typology (*p* <0.001; *d* = 0.44). For the personal variables, significant differences were observed between the typologies in general opposition to equality in SF (*F* = 10.27; *p* < 0.001), with higher scores for the man-favorable typology than for the egalitarian typology (*p* = < 0.001; *d* = 0.44). Group-based dominance in SF (*F* = 12.42; *p* < 0.001) and in SS (*F* = 20.20; *p* < 0.001) was supported with higher scores for the man-favorable typology than for the egalitarian typology (*p* < 0.001; *d* = 0.47 for SF and *p* < 0.001; *d* = 0.55 for SS), and with higher scores for the man-favorable typology than for woman-favorable typology (*p* = 0.003; *d* = 0.35 for SF and *p* < 0.001; *d* = 0.43 for SS). No differences were found in relationship satisfaction as an interpersonal variable. Finally, regarding the social variables, differences were encountered in acceptance of sexual shyness in men in SF (*F* = 5.75; *p* = 0.003) with higher scores for the man-favorable typology than for the egalitarian typology (*p* = 0.002; *d* = 0.31), and in SS (*F* = 7.85; *p* < 0.001) with higher scores for the woman-favorable typology than for the egalitarian typology (*p* = 0.002; *d* = 0.40). See Table 2.

**TABLE 2:** Differences in sexual satisfaction and the variables associated by the sexual double standard adherence types in the sexual freedom (SF) and the sexual shyness (SS) areas in men.

	Typologies of SDS adherence in SF					Typologies of SDS adherence in SS				

			Egalitarian (*n* = 259)	Man-favorable (*n* = 186)	Woman-favorable (*n* = 165)			Egalitarian (*n* = 242)	Man-favorable (*n* = 244)	Woman-favorable (*n* = 123)

	*F*	*p*	*M* (*SD*)	*M* (*SD*)	*M (SD)*	*F*	*p*	*M (SD)*	*M (SD)*	*M (SD)*

Sexual satisfaction	8.41	<0.001	30.01 (5.66)_*a*_	27.64 (5.01)_*a*_	28.98 (5.94)	3.09	0.046	29.62 (5.83)	28.25 (6.55)	28.99 (6.15)
Personal variables										
General opposition to equality	10.27	<0.001	17.08 (7.78)_*a*_	20.69 (8.67)_*a*_	18.72 (8.51)	5.42	0.005	17.51 (7.97)	19.98 (8.52)	18.82 (8.35)
Support for group-based dominance	12.42	<0.001	23.15 (7.86)_*a*_	26.95 (8.17)_*a b*_	24.06 (8.22)_*b*_	20.20	<0.001	22.48 (7.78)_*a*_	27 (8.48)_*a b*_	23.51 (7.77)_*b*_
Sexual excitation	0.89	0.412	15.59 (3.58)	15.85 (3.97)	16.08 (3.78)	1.85	0.159	15.76 (3.82)	15.59 (3.9)	16.38 (3.47)
Sexual inhibition due to the threat of performance failure	4.22	0.015	11.72 (5.59)	10.99 (2.55)	11.44 (2.69)	2.02	0.133	11.59 (2.76)	11.25 (2.64)	11.79 (2.27)
Sexual inhibition due to the threat of performance consequences	1.83	0.161	9.05 (2.62)	8.71 (2.81)	9.27 (3.02)	0.95	0.387	9.22 (2.79)	8.88 (2.70)	9.06 (2.85)

Interpersonal variables										
Relationship satisfaction	3.83	0.022	30.76 (5.37)	29.42 (5.81)	30.67 (4.76)	1.99	0.137	30.91 (5.15)	29.95 (5.54)	30.24 (5.34)

Social variables										
Acceptance of sexual shyness in men	5.75	0.003	2.82 (2.16)_*a*_	3.54 (2.43)_*a*_	3.02 (2.07)	7.85	<0.001	2.64(2.17)_*a*_	3.28 (3.38)	3.5 (2.09)_*a*_
Acceptance of sexual freedom in women	0.67	0.525	6.40 (3.06)	6.27 (2.37)	6.61 (2.84)	0.23	0.791	6.43 (3.16)	3.3 (2.63)	6.49 (2.28)
Acceptance of traditional gender role distribution	2.92	0.058	12.49 (6.06)	13.15 (5.09)	11.68 (5.83)	3.20	0.041	11.9 (6.25)	13.18 (5.57)	12.07 (5.49)

**p-*value threshold α = 0.005. The same subscript letter denote significantly differ between these groups (*p* < 0.005).*

For women, no significant differences were found in sexual satisfaction between the different SDS adherence types in the sexual behavior areas (i.e., SF and SS). Differences appeared for personal variables in general opposition to equality in SS (*F* = 15.97; *p* < 0.001) with higher scores for the man-favorable typology than for the egalitarian typology (*p* < 0.001; *d* = 0.57). Group-based dominance in SS (*F* = 25.51; *p* < 0.001) was supported with higher scores for the man-favorable typology than for the egalitarian typology (*p* < 0.001; *d* = 0.74), and with higher scores for the man-favorable typology than for the woman-favorable typology (*p* < 0.001; *d* = 0.50). For sexual excitation in SS (*F* = 6.18; *p* = 0.002), the man-favorable typology obtained higher scores than the egalitarian typology (*p* = 0.002; *d* = 0.36). No differences were found in relationship satisfaction as an interpersonal variable. Finally among the social variables, differences were encountered in acceptance of sexual shyness in men in the SS (*F* = 8.07; *p* < 0.001), with higher scores for the woman-favorable typology than for the egalitarian typology (*p* = 0.001; *d* = 0.37). See Table 3.

**TABLE 3:** Differences in sexual satisfaction and the variables associated by the sexual double standard adherence types in the sexual freedom (SF) and the sexual shyness (SS) areas in women.

	Typologies of SDS adherence in SF				Typologies of SDS adherence in SS					

			Egalitarian (*n* = 280)	Man-favorable (*n* = 108)	Woman-favorable (*n* = 196)			Egalitarian (*n* = 271)	Man-favorable (*n* = 134)	Woman-favorable (*n* = 166)
	***F***	***p***	***M* (*SD*)**	***M* (*SD*)**	***M (SD)***	***F***	***p***	***M (SD)***	***M (SD)***	***M (SD)***

Sexual satisfaction	1.75	0.175	29.46 (5.96)	28.57 (7.24)	28.37 (7.17)	1.16	0.314	29.25 (6.62)	28.21 (7.28)	29.11 (6.10)

Personal variables										
General opposition to equality	4.48	0.012	15.79 (6.94)	17.95 (8.44)	17.52 (8.24)	15.97	<0.001	15.04 (6.44)_*a*_	19.44 (8.73)_*a*_	17.37 (8.20)
Support for group-based dominance	5.65	0.004	21.10 (7.94)	24.04 (8.7)	22.73 (8.26)	25.51	<0.001	20.35 (7.66)_*a*_	26.28 (8.34)_*a b*_	22.25 (7.78)_*b*_
Sexual excitation	2.95	0.053	16.36 (3.51)	16.79 (3.99)	17.18 (3.65)	6.18	0.002	16.39 (3.64)_*a*_	17.69 (3.47)_*a*_	16.54 (3.67)
Sexual inhibition due to the threat of performance failure	5.7	0.004	10.98 (2.32)	10.16 (2.76)	10.41 (2.35)	3.04	0.049	10.89 (2.45)	10.31 (2.62)	10.48 (2.20)
Sexual inhibition due to the threat of performance consequences	0.57	0.565	8.48 (3.03)	8.17 (3.02)	8.23 (3.05)	0.11	0.896	8.45 (3.01)	8.31 (3.18)	8.38 (2.98)

Interpersonal variables										
Relationship satisfaction	2.2	0.112	30.76 (5.42)	30.55 (5.97)	29.64 (6.43)	0.34	0.709	30.35 (5.61)	30.84 (5.95)	30.5 (5.54)

Social variables										
Acceptance of sexual shyness in men	0.7	0.496	2.9 (2.24)	2.98 (2.32)	3.15 (2.23)	8.07	<0.001	2.60 (2)_*a*_	3.24 (2.47)	3.41 (2.32)_*a*_
Acceptance of sexual freedom in women	0.18	0.837	5.91 (2.72)	5.95 (2.83)	6.06 (2.68)	1.63	0.196	6.05 (2.67)	6.23 (2.95)	5.69 (2.60)
Acceptance of traditional gender role distribution	0.38	0.685	12.86 (7.06)	12.24 (6.01)	12.54 (6.09)	1.19	0.306	12.65 (7.18)	12.09 (5.65)	13.25 (6.07)

**p-*value threshold α = 0.005. The same subscript letter denote significantly differ between these groups (*p* < 0.005).*

### Regression Models

As coefficients can be interpreted analogously to correlation coefficients (0.10 small, 0.30 moderate, and >0.50 large; Cohen, 1992), all the effect sizes were between small and medium, except for satisfaction with the couple’s relationship.

Table 4 presents the regression models of sexual satisfaction in men. In the SF area, the egalitarian typology model explained 60% of the variance of sexual satisfaction (*F* = 93.80; *p* < 0.001). Those variables with predictive power were relationship satisfaction (β = 0.70) and age (β = −0.13). In the man-favorable typology, the model explained 60% of sexual satisfaction (*F* = 199.91; *p* < 0.001) with the variables relationship satisfaction (β = 0.71) and age (β = −0.23). Finally in the woman-favorable typology, the model explained 39% of sexual satisfaction (*F* = 104.36; *p* < 0.001), where relationship satisfaction was the only variable with predictive power (β = 0.62).

**TABLE 4:** Models of multiple regression analysis for sexual satisfaction in men.

SDS areas	Typologies of SDS adherence	Predictors	*B*	*SE*	β	95% CI	*t*	*p*	*R*^2^
SF	Egalitarian	Relationship satisfaction	0.74	0.04	0.70	0.65, 0.82	16.52	<0.001	0.60
		Age	–0.05	0.02	–0.13	−0.08, −0.02	–3.24	0.001	
	Man-favorable	Relationship satisfaction	0.79	0.05	0.71	0.68, 0.90	14.55	<0.001	0.60
		Age	–0.10	0.02	–0.23	−0.13, −0.06	–4.76	<0.001	
	Woman-favorable	Relationship satisfaction	0.78	0.08	0.62	0.63, 0.93	10.22	<0.001	0.39
SS	Egalitarian	Relationship satisfaction	0.77	0.05	0.68	0.68, 0.87	15.43	<0.001	0.53
		Age	–0.08	0.02	–0.20	−0.11, −0.04	–4.52	<0.001	
	Man-favorable	Relationship satisfaction	0.77	0.05	0.65	0.66, 0.88	14.25	<0.001	0.50
		Sexual inhibition due to the threat of performance failure	0.34	0.12	0.14	0.12, 0.57	2.97	0.003	
	Woman-favorable	Relationship satisfaction	0.81	0.07	0.74	0.67, 0.94	12.02	<0.001	0.54

*SF: sexual freedom; SS: sexual shyness; *R*^2^: adjusted *R*-squared value. *p-*value threshold α = 0.005.*

In the SS area, the egalitarian typology model explained 53% of the variance of sexual satisfaction (*F* = 135.86; *p* < 0.001) by relationship satisfaction (β = 0.68) and age (β = −0.20). The man-favorable typology model explained 50% of sexual satisfaction (*F* = 81.08; *p* < 0.001), including relationship satisfaction (β = 0.65) and sexual inhibition due to threat of performance failure (β = 0.14). Finally, the woman-favorable typology model explained 54% of the variation of sexual satisfaction (*F* = 144.52; *p* < 0.001) with relationship satisfaction (β = 0.74) as the only predictor variable.

Table 5 offers the results of the regression models of sexual satisfaction in women. In the SF area, the egalitarian typology model explained 46% of the variance of sexual satisfaction (*F* = 117.29; *p* < 0.001), with the predictor variable relationship satisfaction (β = 0.66). The man-favorable typology model accounted for 49% (*F* = 51.36; *p* < 0.001) with relationship satisfaction (β = 0.66) and age (β = −0.23). Lastly, the predictive model of the woman-favorable typology explained 55% of sexual satisfaction (*F* = 45.71; *p* < 0.001), including relationship satisfaction (β = 0.66) and age (β = −0.18).

**TABLE 5:** Models of multiple regression analysis for sexual satisfaction in women.

SDS areas	Typologies of SDS adherence	Predictors	*B*	*SE*	β	95% CI	*t*	*p*	*R*^2^
SF	Egalitarian	Relationship satisfaction	0.73	0.05	0.66	0.63, 0.82	14.92	<0.001	0.46
	Man-favorable	Relationship satisfaction	0.80	0.08	0.66	0.63, 0.97	9.45	<0.001	0.49
		Age	–0.10	0.03	–0.23	-0.16,-0.04	–3.3	0.001	
	Woman-favorable	Relationship satisfaction	0.73	0.06	0.66	0.62, 0.84	13.18	<0.001	0.55
		Age	–0.08	0.02	–0.18	-0.13,-0.04	–3.65	<0.001	
SS	Egalitarian	Relationship satisfaction	0.84	0.05	0.71	0.75, 0.94	17.16	<0.001	0.54
		Sexual inhibition due to the threat of performance failure	0.34	0.11	0.12	0.11, 0.56	2.99	0.003	
	Man-favorable	Relationship satisfaction	0.80	0.07	0.65	0.65, 0.95	10.72	<0.001	0.52
		General opposition to equality	–0.16	0.05	–0.19	-0.26,-0.06	–3.10	0.002	
		Age	–0.08	0.03	–0.18	-0.13,-0.03	–3	0.003	
	Woman-favorable	Relationship satisfaction	0.68	0.06	0.61	0.55, 0.80	10.6	<0.001	0.46
		Age	–0.08	0.02	–0.21	-0.12,-0.03	–3.46	0.001	

*SF: sexual freedom; SS: sexual shyness; *R*^2^: adjusted *R*-squared value. *p-*value threshold α = 0.005.*

In the SS area, the egalitarian typology model explained 54% of the variance of sexual satisfaction (*F* = 156.03; *p* < 0.001) through relationship satisfaction (β = 0.71) and sexual inhibition due to threat of performance failure (β = 0.12). The man-favorable typology model accounted for 52% of sexual satisfaction (*F* = 47.50; *p* < 0.001) through relationship satisfaction (β = 0.65), general opposition to equality (β = −0.19), and age (β = −0.18). Finally in the woman-favorable typology, the predictive model explained 46% of sexual satisfaction (*F* = 46.80; *p* < 0.001) through relationship satisfaction (β = 0.61) and age (β = −0.21).

## Discussion

The study objective considers different SDS adherence types in two sexual behavior areas (sexual freedom and sexual shyness) to examine, according to the Ecological Theory (Bronfenbrenner, 1994), the relation of sexual satisfaction with the personal (age, social dominance orientation, propensity for sexual excitation/inhibition), interpersonal (relationship satisfaction) and social (gender norms about sexual behaviors) variables. In order to overcome the limitations of the relation between sexual satisfaction and the SDS, measures of the SDS (Álvarez-Muelas et al., 2020a) and sexual satisfaction (Sánchez-Fuentes et al., 2015) were resorted to, with clear evidence for validity in the Spanish population. Moreover, according to the findings obtained and the recommendations made in previous studies (Álvarez-Muelas et al., 2020b), SDS adherence types in two sexual behavior areas (sexual freedom and sexual shyness) were considered to be cluster factors to analyze both the differences in the pattern of responses to the personal, interpersonal and social variables (i.e., RQ1), and the predictive role of these variables in sexual satisfaction (i.e., RQ2).

To answer RQ1, differences were observed in sexual satisfaction, personal, and social variables by SDS adherence types in men and women and in both sexual behavior areas with a medium effect size. In sexual satisfaction, differences were found for SDS adherence types in men for the SF area, with higher scores for the egalitarian typology than for the man-favorable typology. It would support the notion that sexual satisfaction in men was less when they supported the man-favorable SDS (Haavio-Mannila and Kontula, 2003; Santos-Iglesias et al., 2009), and was favored by the egalitarian posture (Haavio-Mannila and Kontula, 2003). In the Interpersonal Exchange Model of Sexual Satisfaction context (Lawrance and Byers, 1995), the benefits and costs of sexual relationships, along with the non-sexual aspects of the couple’s relationship, played a key role in explaining sexual satisfaction (Sánchez-Fuentes and Santos-Iglesias, 2016). The man-favorable typology promoted men’s proactivity, although the predominant role played by men during sexual activity could imply that they do not report sexual satisfaction (Dworkin and O’Sullivan, 2005) because they could perceive women’s passive role as lack of sexual interest (Fischer et al., 2020). This could lead them to not carry out and meet their expectations in sexual relationships, which could be taken as a cost and would imply less sexual satisfaction. Indeed women conforming, in line with their traditional role, would not cushion these possible consequences for sexual satisfaction (Sánchez et al., 2012). Moreover, the greater support of exercising sexual freedom for men and women that is observed in western societies (Bianchi et al., 2000; Paul et al., 2000; García et al., 2012) could justify that evidence for this association takes place in the SF area.

Differences were also found in some personal variables: general opposition to equality, support for group-based dominance, and sexual excitation. In the social dominance orientation dimensions, higher scores were obtained in the man-favorable typology in men for both sexual areas and in women in the SS area. These results corroborate that both the men and women who support the SDS that favors men (i.e., man-favorable typology) also agree more than egalitarian people with a hierarchical gender social structure (Sidanius and Pratto, 1999). Furthermore, the SDS adherence types seemed to be associated with the sexual excitation in women in the SS area, with higher scores in the man-favorable typology compared to the egalitarian typology. The highest scores for these typologies might reflect that the more conventional socialization of gender roles would seem relevant for propensity of sexual excitation in relation to the domain that promotes recognizing and approving more decorum, chastity, and continence.

Finally in the social variables, it was found that people with different SDS adherence types differed as to how they perceived social gender norms about sexual behavior. Specifically, the acceptance of sexual shyness in men differs significantly among the SDS adherence typologies, with lower scores in the egalitarian typology in men in both areas, and in women in the SS area. The fact that egalitarian men and women (vs. man-favorable men and woman-favorable women) perceived that society does not support sexual shyness in men that much could work as a way to gain validation and support for their most democratic attitude toward the SDS (Guadagno and Cialdini, 2010). Future research should consider the role of social factors and a macropsychological approach to understand the prevalence of sexual attitudes and behaviors.

With RQ2, differences were observed in the predictive variables of sexual satisfaction according to SDS adherence types, the sexual behavior area (SF and SS) and gender. First of all, one of the personal variables that appeared more in the regression models was age, and for both men and women. Age negatively predicted sexual satisfaction, which coincided with the results reported in previous studies (Tren and Schaller, 2010; De Ryck et al., 2012; Sánchez-Fuentes and Sierra, 2015; Træen et al., 2017; Wyverkens et al., 2018). Sexual interest diminished as age increased (Gott and Hinchliff, 2003), as did frequency of sexual activity, while sexual dysfunctions increased (Addis et al., 2006; Sierra et al., 2012; Arcos-Romero and Sierra, 2019). All these sexuality aspects have been associated with sexual satisfaction (Badcock et al., 2014; Sánchez-Fuentes et al., 2014, 2016; Thomas et al., 2015; Calvillo et al., 2018, 2020a). Nevertheless, the weight of age in explaining sexual satisfaction was far from significant, and was not relevant for any cases of the men with the woman-favorable typology in both areas, and with the man-favorable typology in SS, nor for the women with the egalitarian typology in SF and SS. Future research should specifically study the extent to which the negative effect that age has on sexual satisfaction is consistent or, if conversely, internalizing more the egalitarian typology in women or making the hegemony of one gender category more sensitive over another in men can minimize or eliminate the negative effect that age has on men and women’s sexual satisfaction.

Moreover, general opposition to equality also negatively predicted sexual satisfaction in women; specifically, those women with the man-favorable SDS adherence typology in SS area. Within the theory of system justification framework (Jost and Banaji, 1994), the beliefs that justify or rationalize existing inequalities perform a “palliative function,” and are associated with better subjective well-being and physical health (Jost, 2019). However, the assumptions of the aforementioned theory must be qualified depending on the status of the group to which the person belongs. As women belong to the disadvantaged group in the social hierarchy established according to gender, thus when women with a man-favorable SDS adherence typology accept that hierarchy is legitimate, they might internalize their status inferiority with respect to men, which would harm their sexual satisfaction (Lick et al., 2013).

Unlike previous evidence (Lykins et al., 2012; Moyano and Sierra, 2014; Arcos-Romero and Sierra, 2020), sexual inhibition due to threat of performance failure positively predicted sexual satisfaction. Instead no evidence was found for the predictive capacity of sexual excitation and (Lykins et al., 2012) and inhibition due to threat of performance consequences (Moyano and Sierra, 2014). The predictive power of propensity inhibition due to threat of performance failure was both low and variable among adherence typologies according to gender in the SS area. Inhibition due to threat of performance failure promoted sexual satisfaction in men for the man-favorable typology, and also for women in the egalitarian typology. In the SS area, the fact that the men who encouraged their own group’s privileges through women’s sexual shyness could mean that they considered a heavier weight for themselves. This could involve placing more emphasis on sexual activity with a positive impact on sexual satisfaction. For women, the egalitarian typology establishes the same criteria for evaluating sexual behavior when done by oneself or one’s partner. Given this equality, concerns such as sexual performance or the ability to please one’s partner appear to increase, which could have a positive impact on sexual satisfaction. Finally, sexual inhibition is considered to be an adaptation mechanism that hinders sexual response until a stimulus or the sexual situation is evaluated as not being threatening (Granados et al., 2020). These results support the role of gender roles, insofar as socialization, and learning processes could be responsible for the inhibition of gender differences (Pinxten and Lievens, 2016). So future research could consider if these evaluations made in the sexual shyness context could be taken as not being threatening depending on different forms of individual SDS adherence by men or women.

The interpersonal variable relationship satisfaction was the main predictor of sexual satisfaction in all the models, and was the only predictor in the woman-favorable typology in men in both sexual behavior areas and in the egalitarian typology in women in SF area, which confirms the results published in former studies (Byers, 2005; Sánchez-Fuentes et al., 2015; Sánchez-Fuentes and Santos-Iglesias, 2016; Calvillo et al., 2020a). As relationship satisfaction is a component of the Interpersonal Exchange Model of Sexual Satisfaction (Lawrance and Byers, 1995), higher relationship satisfaction levels imply more sexual satisfaction for partners (Haavio-Mannila and Kontula, 2003; Byers, 2005). This study provides evidence that relationship satisfaction was the variable that more clearly explains sexual satisfaction in different forms of adherence to SDS and in both sexual behavior areas.

No predictive role was found in sexual satisfaction with gender norms about sexual behaviors. Systematic reviews performed to explain sexual satisfaction with the Ecological Theory Model have found that the predictive power of social variables is poor, and stressed that personal and interpersonal variables are the main predictors of sexual satisfaction (Sánchez-Fuentes et al., 2014; Calvillo et al., 2018). Previous research works have also demonstrated that the impact of gender norms on sexuality would be mediated by the pressure that someone is under to adapt to these norms (Sánchez et al., 2005). Accordingly, the fact that gender norms have no predictive effect on sexual satisfaction might be because we did not measure adherence to these norms, rather the interviewee’s perception of society adhering to these gender norms about sexual behaviors.

This study has its limitations, which influence how its results are interpreted. Our sample was selected by performing non-probabilistic sampling of the Spanish population, which included individuals with a heterosexual orientation. So the extent to which these results can be generalized to the population and other sexual orientations must be taken into account. Given the use of explicit SDS measures, future studies could contemplate implicit measuring (Endendijk et al., 2020; Thompson et al., 2020), as well as the couple’s dyad and the role that the variables applied to the couple’s relationship play (e.g., time the relationship lasts, living together) to study sexual satisfaction in the couple context. Likewise, we stress the cross-sectional descriptive nature of this study, and recommend analyzing these results by a quasiexperimental methodology.

## Conclusion

To conclude, our results contribute to the study of sexual satisfaction for several reasons. We observed higher sexual satisfaction levels in the egalitarian typology to evaluate sexual behaviors, which means that the postures that defend the men or women hegemony would appear to make sexual satisfaction difficult. Particularly in the sexual freedom area, the men belonging to the man-favorable typology would experience less sexual satisfaction than the men of the egalitarian typology. It was found that the differences in sexual satisfaction and related variables (i.e., personal, interpersonal, and social nature) depended on both the person’s characteristics, such as gender and SDS adherence type, and also on the sexual behavior area (i.e., sexual freedom and sexual shyness) referred to by the attitude toward the SDS. Moreover, and once again, this study supports applying the Ecological Theory (Bronfenbrenner, 1994) to propose the sexual satisfaction prediction. We generally obtained more predictor variables of sexual satisfaction in women than in men. We highlight that in all explanatory models, a variable of an interpersonal nature was the main predictor of sexual satisfaction: relationship satisfaction. Our study also evidenced the role of the personal variables: age, propensity for sexual inhibition, general opposition to equality. The predictive capacity of personal and interpersonal factors have on sexual satisfaction depends on the characteristics of the person, such as gender and the type of adherence to the SDS, and the sexual behavior areas (i.e., freedom and shyness) referred to by the attitude of the double standard. The age variable turned out to be predictive in more models. Propensity for sexual inhibition due to threat of performance failure and general opposition to equality were found in the sexual shyness area, which means more variables predicting sexual satisfaction in this domain. Finally, the presented results could contribute to the clinical field if we consider the interiorization of gender roles in the sexual behavior area as a variable to bear in mind. In this way, these findings could be considered in sexual health programs through the support of egalitarian norms about sexual behavior to foster satisfactory and pleasant sexuality. Moreover, they could contribute to sexual satisfaction in sexual therapy in the couple context by promoting positively related variables and inhibiting those that are negatively associated with such therapy to improve heterosexual couple’s sexual health. In this way, sexual inhibition due to the threat of performance failure has a role positive in sexual satisfaction in the sexual shyness area, whose interpretation and intervention depends on SDS adherence types and gender.

## Data Availability Statement

The raw data supporting the conclusions of this article will be made available by the authors, without undue reservation.

## Ethics Statement

The studies involving human participants were reviewed and approved by The Ethics Committee on Human Research of the University of Granada in Spain. The patients/participants provided their written informed consent to participate in this study.

## Author Contributions

AÁ-M: data curation, formal analysis, methodology, software, resources, investigation, original draft preparation, writing, review, and editing. CG-B: conceptualization, formal analysis, project administration, resources, investigation, original draft preparation, writing, review, and editing. JS: conceptualization, funding acquisition, project administration, supervision, investigation, original draft preparation, writing, review, and editing. All authors contributed to the article and approved the submitted version.

## Conflict of Interest

The authors declare that the research was conducted in the absence of any commercial or financial relationships that could be construed as a potential conflict of interest.
